# Photodegradation of Amoxicillin in Aqueous Systems: A Review

**DOI:** 10.3390/ijms25179575

**Published:** 2024-09-04

**Authors:** Mohammad Ashraf Ali, Ibrahim M. Maafa

**Affiliations:** Department of Chemical Engineering, College of Engineering and Computer Sciences, Jazan University, Jazan 45142, Saudi Arabia; mashrafali@jazanu.edu.sa

**Keywords:** photolysis, photodegradation, photocatalysis, TiO_2_-based catalysts, non-TiO_2_-based catalysts, aqueous system, nanomaterials

## Abstract

Amoxicillin (AMX) is utilized in the treatment of several infectious diseases, and its concentration in wastewater has increased quite significantly over the years, posing high health hazards for humans and other living organisms. Investigations are in progress globally to eliminate AMX and other related pollutants using several methods that include adsorption, photolysis, photocatalytic degradation, photoelectrocatalytic degradation, and electrochemical conversion. AMX can be eliminated efficiently from the environment using photodegradation, either by photolysis or a photocatalytic process. Several types of semiconductor NMs have been used to eliminate AMX and other related drugs present in wastewater. This review spans the photodegradation studies conducted during the years 2018–2024 to degrade and eliminate AMX in aquatic systems. Several studies have been reported to eliminate AMX from different water streams. These studies are categorized into TiO_2_-containing and non-TiO_2_-based catalysts for better comparison. A section on photolysis is also included, showing the use of UV alone or with H_2_O_2_ or PS without using any nanomaterial. A tabulated summary of both types of catalysts showing the catalysts, reaction conditions, and degradation efficiency is presented. Researchers have used a variety of reaction conditions that include radiation types (UV, solar, and visible), pH of the solution, concentration of AMX, number of nanomaterials, presence of other additives and activators such as H_2_O_2_ as oxidant, and the influence of different salts like NaCl and CaCl_2_ on the photodegradation efficiency. TiO_2_ was the best nanomaterial found that achieved the highest degradation of AMX in ultraviolet irradiation. TiO_2_ doped with other nanomaterials showed very good performance under visible light. WO_3_ was also used by several investigators and found quite effective for AMX degradation. Other metal oxides used for AMX elimination were derived from molybdenum, zinc, manganese, copper, cerium, silver, etc. Some researchers have used UV and/or visible irradiation or sunlight, without using solid catalysts, in the presence of oxidants such as H_2_O_2_. A summarized description of earlier published reviews is also presented.

## 1. Introduction

Researchers worldwide are attempting to remove organic contaminants from wastewater using a variety of strategies, including adsorption, photolysis, photocatalysis, photoelectrocatalysis, and electrochemical conversion. These organic contaminants include dyes, medicines, and surfactants, which have been found in many aquatic environments worldwide. These organic pollutants pose toxicity to aquatic and land-dwelling creatures. The conventional treatment processes for wastewater eliminate these substances only partly, and they generally remain in trace concentrations in treated effluent [1]. Photocatalysis is one of the best techniques for the complete elimination of organic pollutants from wastewater. Organic dyes such as methylene blue, crystal violet, rhodamine B, methyl orange, alizarin red, Eriochrome Black T indicator, reactive red 250, and Congo red have been photodegraded successfully using different types of nanocomposites, which include Co-SnO_2_-loaded, sulfur-doped graphitic carbon nitride [2]; spinel MOF [3]; ZnO [4]; reactive red 250 [5]; Ag/Mn–ZnO [6]; Ni/g-C_3_N_4_/ZnO [7] and Mn/g-C_3_N_4_/ZnO [4]; Ba-doped Mg ferrites [8]; and WO_3_/NiWO_4_ [9]. Similarly, pharmaceuticals present in wastewater have been photodegraded by several researchers using a variety of nanomaterials. Husain et al. successfully degraded ibuprofen and ofloxacin in wastewater using the photo-Fenton process with MnO_2_ and ozonation, achieving over 90% elimination [10]. 

## 2. Reported AMX Photodegradation Studies

One of the most-used antibiotics is AMX for the treatment of urinary, gastrointestinal, skin, and respiratory bacterial infections [11]. The World Health Organization has ranked AMX as one of the medically important antimicrobial medicines for human use, and this ranking has encouraged higher AMX use that has led to increased exposure of AMX in the environment [12]. Several studies have been reported to eliminate AMX from wastewater streams. We have categorized the studies into TiO_2_-containing and non-TiO_2_-based catalysts for a clear understanding.

### 2.1. TiO_2_-Containing Catalysts

Several researchers have used TiO_2_ alone or TiO_2_ doped with metals and mixed with metal oxides and carbon nitrides. These studies are reported here. At the end of this section, Table 1 presents a summary of the performance of some of the TiO_2_-based nanomaterials (NMs) and nanocomposites used for the photodegradation of AMX.

Alshandoudi et al. investigated the efficiency of AMX removal through photocatalytic degradation using nanoTiO_2_ and nanoTiO_2_/nanohydroxyapatite composites and achieved 85.3 and 99.5% degradation, respectively, within 90 min using catalyst concentrations of 0.9 g/L [13]. 

Lalliansanga et al. investigated Ce^3+^/TiO_2_ thin-film photocatalysts to degrade tetracycline and AMX under UVA irradiation [14]. The presence of Ce^3+^ was found to be quite significant to enhance photocatalytic degradation of the antibiotics. 

Mhemid, et al. performed photodegradation of AMX using TiO_2_ and N-doped TiO_2_ in an aqueous solution under solar irradiation [15]. The N-doped TiO_2_ was synthesized from urea by the sol-gel process. The optimum conditions achieved were pH 5 and H_2_O_2_ concentration of 400 mg/L, using 50 mg catalyst and AMX concentration of 25 mg/L, for a photodegradation efficiency of 95.8% with N-TiO_2_ compared with 89.3% for TiO_2_. 

**Figure 1:**
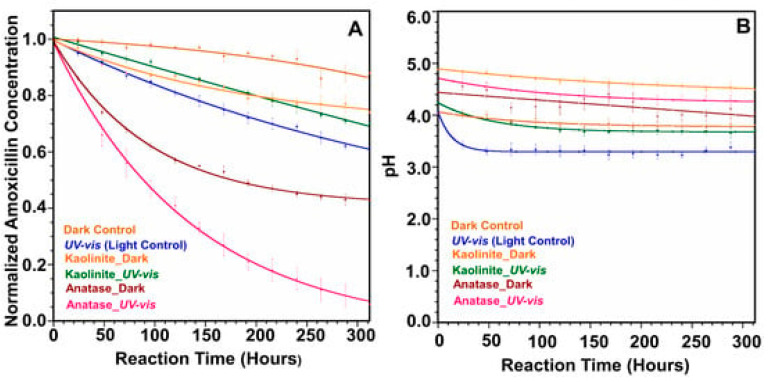
A comparison of AMX degradation under different experimental conditions. (**A**) Degradation plots of AMX with TiO_2_ anatase and kaolinite under light and dark conditions. (**B**) pH variation of the AMX as a function of reaction time [16].

**Figure 2:**
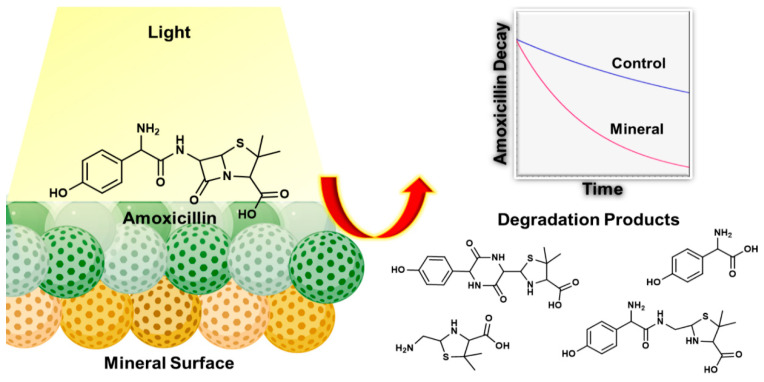
Interaction of AMX with the mineral surface and degradation into simpler products [16].

**Figure 3:**
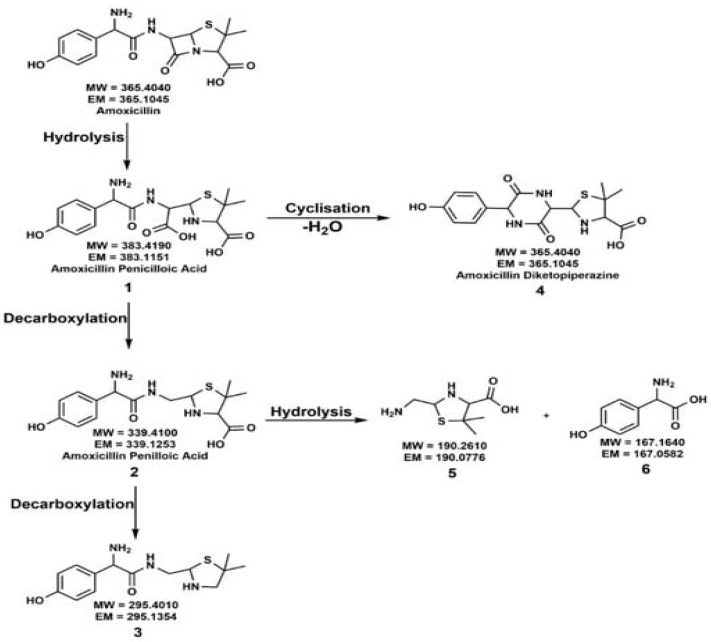
Proposed degradation reactions of AMX under dark and solar radiation without minerals [16].

Ellepola et al. investigated a TiO_2_ anatase for AMX photodegradation and observed AMX degradation increased by 4.5-fold in the presence of light [16]. Figure 1 shows AMX degradation plots with TiO_2_ anatase and kaolinite under dark and irradiation, and the pH variation of the AMX, as a function of reaction time. Almost complete degradation of AMX was achieved using TiO_2_ anatase under UV–Vis irradiation in 300 h. Figure 2 shows how the AMX interacted with the mineral surface in the presence of light and degraded into simpler products. Figure 3 shows the proposed degradation of AMX into different compounds under dark and solar radiation in the absence of minerals. 

**Figure 4:**
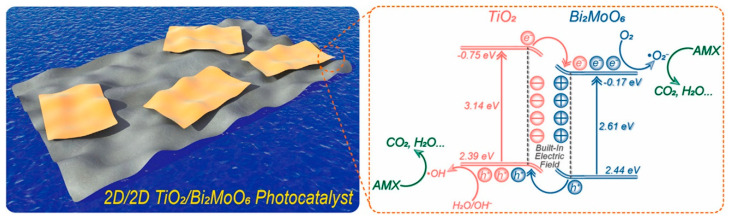
Visualization of 2D/2D TiO_2_/Bi_2_MoO_6_ catalyst and AMX photodegradation process [17].

Wang et al. prepared 2D/2D TiO_2_/Bi_2_MoO_6_ material into nanosheets that showed high effectiveness and steady performance for AMX photodegradation, which was 18.2 and 5.7 times higher than TiO_2_ and Bi_2_MoO_6_, respectively [17]. Figure 4 presents a visualization of the 2D/2D TiO_2_/Bi_2_MoO_6_ photocatalyst and the process of AMX photodegradation using TiO_2_/Bi_2_MoO_6_ nanomaterials.

Zamani et al. described a visible-light-activated Ag/Ag_2_O/TiO_2_ heterostructure photocatalyst for the degradation of AMX [18]. The results revealed that the greatest AMX photodegradation achieved was 97.9% within 80 min at pH 6 with an initial AMX content of 20 mg/L.

Aissani et al. developed a supported catalyst TiO_2_/Mg-Fe-LDH and used it for the photodegradation of AMX under UVA irradiation in basic conditions [19]. The catalyst exhibited excellent photodegradation efficiency, and high recyclability was retained after four cycles. 

Hussien et al. synthesized core/shell g-C_3_N_4_@TiO_2_ heterojunction using melamine pyrolysis and used it for AMX photodegradation under visible light irradiation [20]. The photocatalyst g-C_3_N_4_@TiO_2_ exhibited 100% degradation of AMX in 20 min. The AMX photodegradation using g-C_3_N_4_@TiO_2_ was three times higher compared with g-C_3_N_4_ under simulated solar irradiation.

Gao et al. synthesized a ternary Ag/TiO_2_/M-g-C_3_N_4_ nanocomposite that showed the highest degradation efficiency for AMX compared with its components [21]. 

**Figure 5:**
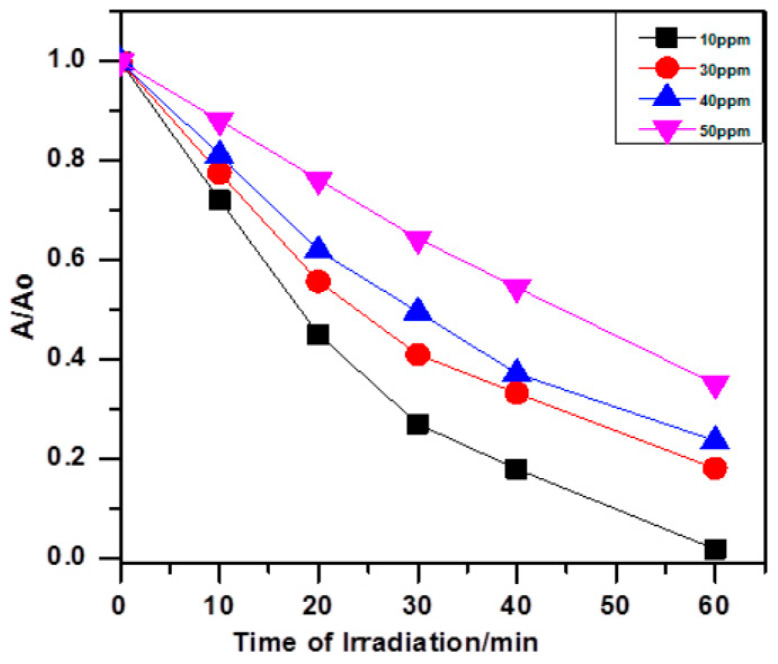
Photocatalytic degradation of AMX at different initial concentrations of 10 to 50 ppm using 0.2 g/L of catalyst at 25 °C [22].

Alkaim et al. [22] performed photodegradation of AMX over TiO_2_ under UV irradiation and achieved 98.0% removal after one hour using an initial AMX concentration of 10 mg/L at 25 °C and 0.2 g/L of the catalyst. Higher initial AMX concentrations experienced lower degradation efficiencies, as shown in Figure 5. 

Salimi et al. investigated the photodegradation of AMX with Pt and Bi co-doped TiO_2_ photocatalysts. Among the developed photocatalysts, 1Pt-5Bi-TiO_2_ photodegraded AMX at 87.7% under visible light irradiation [23]. 

Yılmaz et al. produced co-doped TiO_2_ and utilized it to photodegrade AMX in an aqueous solution using UVC and visible irradiation. AMX was completely degraded during 4 h of UVC irradiation and 5 h of visible irradiation [24]. 

Bergamonti et al. evaluated TiO_2_-supported chitosan scaffolds (TiO_2_/CS) for AMX photodegradation under UV–Vis irradiation [25]. TiO_2_ chitosan scaffolds were prepared by 3D printing through dispersion of 6.0% *w*/*v* chitosan and 1.0% *w*/*v* commercial P25-TiO_2_ in powder form. The TiO_2_/CS system showed a high recycling photodegradation efficiency. 

Chinnaiyan et al. performed photodegradation of AMX-containing synthetic hospital wastewater using a 200 mL photoreactor, TiO_2_ as a photocatalyst, and UV irradiation at 365 nm using a 125 W mercury vapor lamp [26]. The maximum AMX degradation achieved was 90.0% at pH 7.6, using a TiO_2_ dosage of 563 mg/L and an initial concentration of 10 mg/L within 150 min irradiation. 

**Figure 6:**
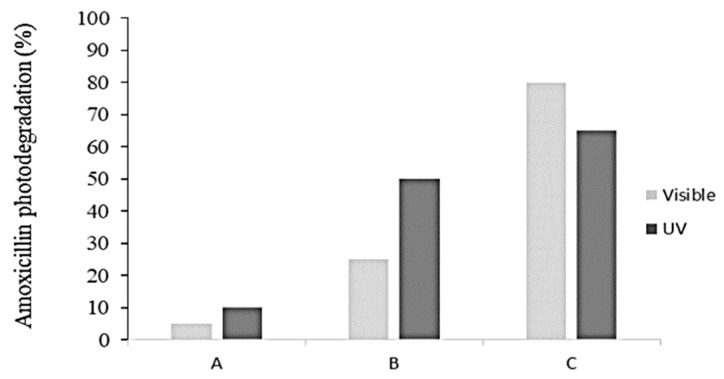
AMX photodegradation efficiency under UV and visible light. (A) No photocatalyst, (B) TiO_2_, and (C) Cu-TiO_2_. (AMX solution volume 100 mL, AMX concentration 10 mg/L, photocatalysts 40 mg, irradiation time 24 h, and pH 6) [27].

Wahyuni et al. synthesized Cu-TiO_2_ and used it under visible light irradiation for AMX degradation in water [27]. It was found that 90% of 10 mg/L AMX was degraded using 0.40 g/L of the Cu-TiO_2_ photocatalyst (having 4.56 mg Cu/g of TiO_2_) after 24 h at pH 6. Figure 6 shows the AMX photodegradation efficiency under UV and visible light using 100 mL AMX solution having 10 mg/L concentration after 24 h of irradiation at pH 6. In the case of TiO_2_, UV irradiation produced more degradation than visible radiation, while for Cu-TiO_2_, visible light irradiation produced more degradation than UV radiation.

**Figure 7:**
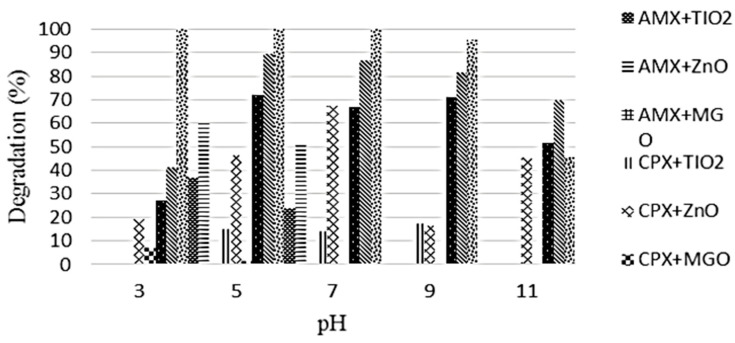
Effect of pH on the degradation efficiency of AMX and cephalexin (CPX) under UV irradiation using ZnO, TiO_2,_ and GO-Fe_3_O_4_ catalyst. Irradiation time 20 min, catalyst 1 g/L, antibiotics 5 mg/L, UV radiation intensity 6 W [28].

**Figure 8:**
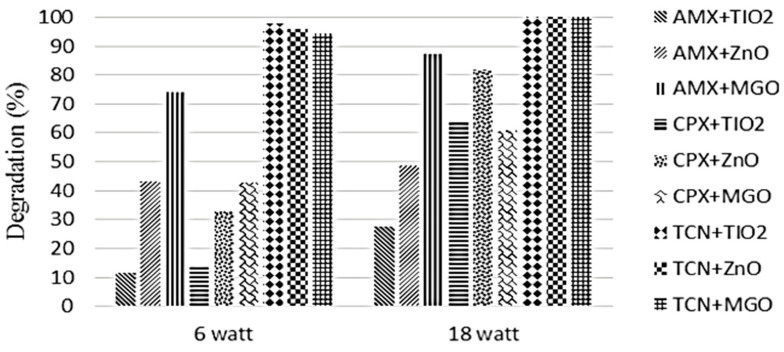
Effect of radiation intensity on the degradation efficiency of AMX, cephalexin (CPX), and tetracycline (TCN) under UV irradiation using ZnO, TiO_2,_ and GO-Fe_3_O_4_ catalysts: pH (AMX, CPX, TCN with GO-Fe_3_O_4_) unset; pH (AMX, CPX, TCN with TiO_2_ or ZnO) 5, 7, 5; time (AMX, CPX, and TCN/MGO) 45, 45, 15 min; time (AMX, CPX, and TCN/TiO_2_ or ZnO) 15, 45, 45 min; MGO quantity (with AMX, CPX, or TCN) 4, 4, 2 g/L; TiO_2_ or ZnO quantity (with AMX, CPX, or TCN) 2, 2, 2 g/L; antibiotics concentration 15 mg/L [28].

Fazilati used TiO_2_, ZnO, and GO-Fe_3_O_4_ for AMX photodegradation under UV irradiation and achieved maximum removal efficiency of 27.6%, 48.6%, and 87.1%, respectively, under the optimum conditions of AMX 15 mg/L at 18 W of UV irradiation [28]. It was concluded that using the catalyst GO-Fe_3_O_4_ under UV irradiation, the solution pH did not affect the photodegradation efficiency of AMX (Figure 7). Figure 8 shows that the photodegradation of AMX increased from 6 W to 18 W using TiO_2_, ZnO, and GO-Fe_3_O_4_ catalysts. For TiO_2_ and GO-Fe_3_O_4_, more photodegradation was observed than with ZnO while increasing from 6 W to 18 W.

**Figure 9:**
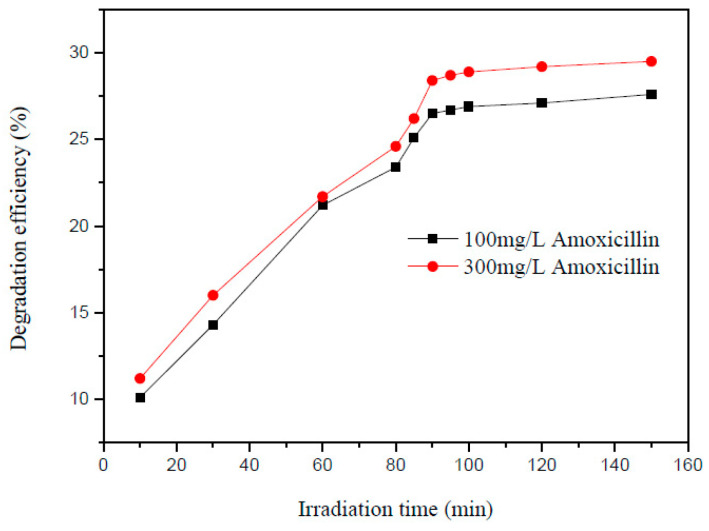
Effect of initial AMX concentrations on the degradation efficiency for 200 mL solution containing 100 mg/L and 300 mg/L AMX with 0.5 g CaTiO_3_ at room temperature at different irradiation times [29].

**Figure 10:**
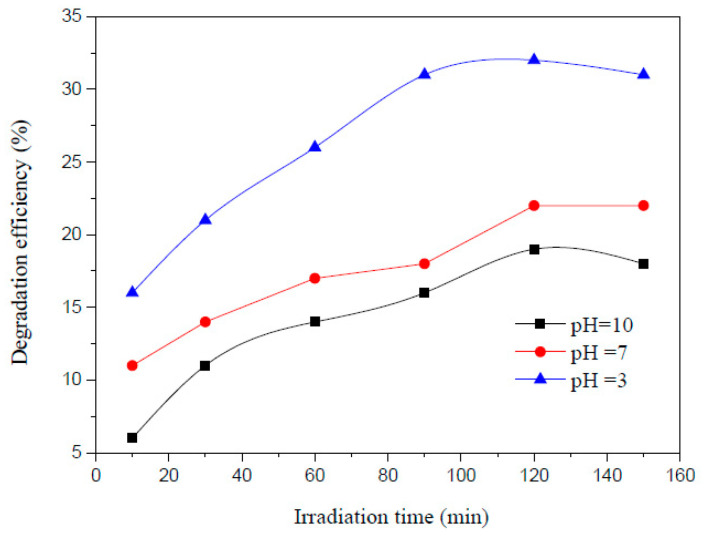
AMX degradation efficiency of 200 mL solution containing 100 mg/L AMX with 0.5 g CaTiO_3_ at room temperature at different irradiation times at pH 3, 7, and 10 [29].

**Figure 11:**
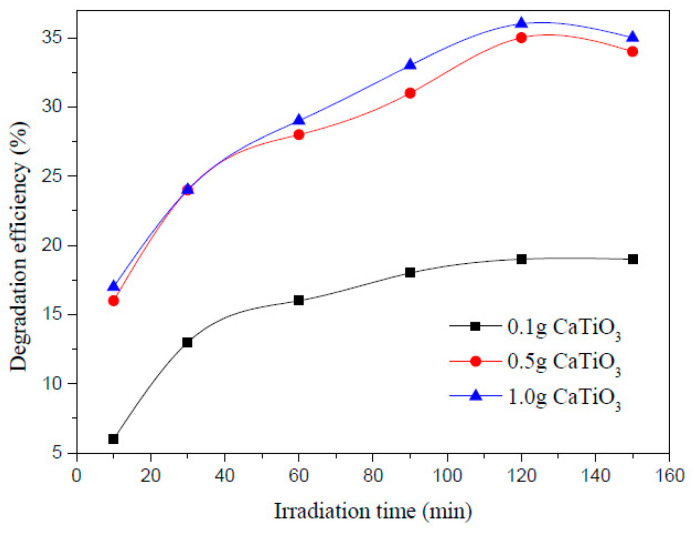
Effect of CaTiO_3_ amount on the AMX degradation efficiency for 200 mL aqueous solution having 100 mg/L AMX at pH 3 and room temperature [29].

**Figure 12:**
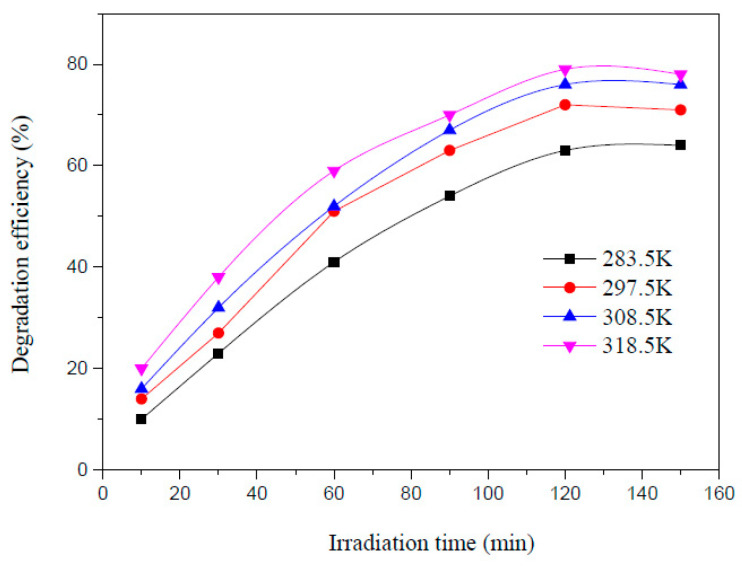
Effect of temperature on the AMX degradation efficiency for 200 mL solution having 100 mg/L AMX with 0.5 g CaTiO_3_ and 0.058 g NaCl at pH 3 [29].

Zhou et al. carried out photoelectrocatalytic degradation of AMX in water successfully and achieved maximum degradation of 79% using 200 mL AMX solution having 100 mg/L concentration with 0.5 g CaTiO_3_, 0.058 g NaCl added as the electrolyte at pH 3, and 45 °C for 120 min irradiation [29] (Figure 9). Using the photocatalytic process, the degradation efficiency was 35.8% only. Figure 10 shows the degradation efficiency for a 200 mL AMX solution with a 100 mg/L concentration and 0.5 g CaTiO_3_ at room temperature at pH 3, 7, and 10. The highest degradation was observed at an acidic pH of 3, which is contrary to the other reported research, and it could be due to the presence of CaTiO_3_. Figure 11 exhibits the effect of CaTiO_3_ concentration on the AMX degradation in 200 mL aqueous solution having 100 mg/L AMX at pH 3 and room temperature. A higher concentration of CaTiO_3_ produced a higher degradation of AMX. Figure 12 shows the effect of temperature on degradation efficiency for 200 mL aqueous solution having 100 mg/L AMX with 0.5 g CaTiO_3_ and 0.058 g NaCl at pH 3 with a current intensity of 0.03 mA/cm^2^. Higher degradation was observed by increasing the temperature. The photoelectrocatalytic degradation data fit the first-order kinetics.

**Figure 13:**
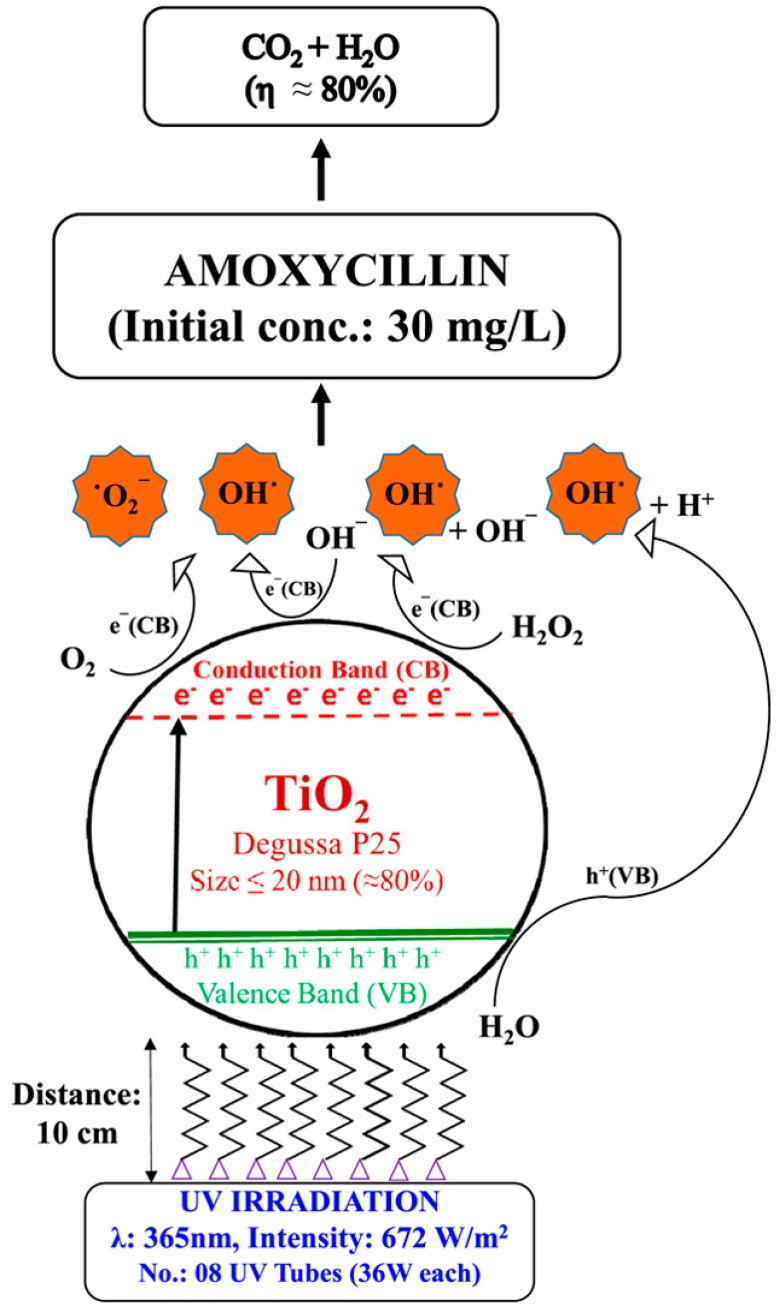
Process of AMX degradation using TiO_2_ under UV irradiation (UVA 365 nm, 672 W/m^2^) for 30 mg/L AMX concentration, TiO_2_ dosage of 450 mg/L, H_2_O_2_ concentration of 150 mg/L at pH 7.0 [30].

Verma et al. investigated AMX degradation using TiO_2_ photocatalysis in aqueous solution using UVA (365 nm) and sunlight [30]. The optimal conditions to achieve 80% AMX degradation were a TiO_2_ dosage of 450 mg/L, an AMX concentration of 30 mg/L, an H_2_O_2_ concentration of 150 mg/L, and pH 7.0 under UV irradiation (672 W/m^2^). Substantial enhancement in the degradation rate was observed using H_2_O_2_ and sonication (40 KHz), but the maximum degradation remained the same (Figure 13). 

**Figure 14:**
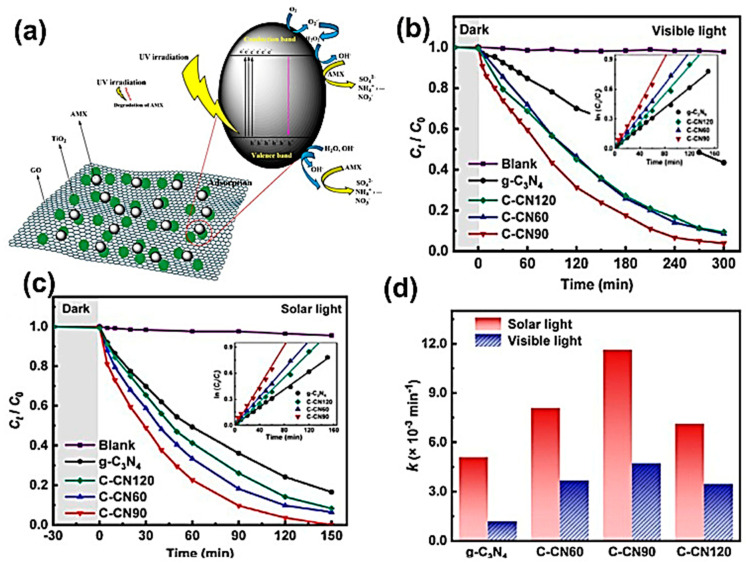
(**a**) Possible mechanism of AMX degradation at the GO/TiO_2_ surface [31]. Photocatalytic degradation kinetics of AMX by the synthesized materials under (**b**) visible light and (**c**) simulated solar light. (**d**) AMX degradation rate constants under solar and visible light [32].

Balarak et al. reported performing AMX photocatalytic degradation under UV irradiation using TiO_2_ NPs loaded on graphene oxide (GO/TiO_2_) [31]. The AMX degradation efficiency was almost 100% at pH 6, using a GO/TiO_2_ concentration of 0.4 g/L and AMX concentration of 50 mg/L, at UV irradiation intensity of 36 W. The catalyst showed exceptional recyclability for four repeated cycles. The detection of NH_4_^+^, NO_3_^−^, and SO_4_^2−^ ions confirmed good mineralization of the AMX. Figure 14a illustrates the possible mechanism of AMX degradation at the GO/TiO_2_ surface. 

Huang et al. prepared novel carbon-rich g-C_3_N_4_ nanosheets with large surface areas, which showed superior photocatalytic activity for AMX degradation under solar light [32], as shown in Figure 14b–d. The best degradation results were obtained using the catalyst C-CN90, which was prepared by pyrolysis of a mixture of urea and 90 mg of 1,3,5-cyclohexanetriol for the optimum composite.

Table 1 lists the performance of TiO_2_-based nanomaterials (NMs) and nanocomposites used for photodegradation of AMX and their reaction conditions. The titania-based catalysts consist of TiO_2_ NPs and TiO_2_ doped with other active metals or mixed with other metal oxides. In the case of pure TiO_2_, UV radiation was used in most of the studies. When mixed with other metal oxides and doped with other metals, visible radiation was employed preferably, due to the reduction in the bandgap of the catalysts as a result of interaction between TiO_2_ and other metals and metal oxides.

**Table 1:** Performance of TiO_2_-based nanomaterials (NMs) and nanocomposites used for photodegradation of AMX.

No	Catalyst Type	Process Conditions	Degradation Efficiency, %	Degradation Time, h	Ref.
1.	TiO_2_ NPs, 0.2 g/L	UV light irradiation, AMX 10 mg/L	98.0%	1	[22]
2.	TiO_2_ NPs, 450 mg/L	UV light irradiation (672 W/m^2^), AMX 30 mg/L, H_2_O_2_ (30% *w*/*v*) 150 mg/L, pH 7.0	80	4	[30]
3.	TiO_2_ NPs, 563 mg/L	UV irradiation at 365 nm (125 W Hg vapor lamp), 200 mL photoreactor, pH 7.6, AMX 10 mg/L	90	2.5	[26]
4.	TiO_2_ NPs, 0.9 g/L	UV light irradiation	85.3	1.5	[13]
5.	TiO_2_ NPs, 40 mg	Visible light irradiation, AMX 10 mg/L, 100 mL solution, pH 6	50	24	[28]
6.	N-TiO_2,_ 50 mg	Solar light irradiation, H_2_O_2_ 400 mg/L, pH 5, AMX 25 mg/L	95.8%	1	[15]
7.	nanoTiO_2_/nano-hydroxyapatite composite, 0.9 g/L	UV light irradiation	99.5	1.5	[13]
8.	g-C_3_N_4_@TiO_2_	Visible light irradiation	100%	0.33	[20]
9.	Ag/Ag_2_O/TiO_2_	Visible light irradiation, AMX 20 mg/L, pH 6	97.9%	1.33	[18]
10.	Cu-TiO_2_ NPs, 0.40 g/L	Visible light irradiation, AMX 10 mg/L, 100 mL solution, pH 6	90	24	[28]
11.	10%Cu_2_O/TiO_2_ nanotubes, 1.5 g/L	Visible light irradiation, AZT 100 μg/mL, pH 7	100	1.5	[33]
12.	Zn-doped TiO_2_	UV light irradiation, H_2_O_2_ 550 mL/L, pH 3	95	1.5	[34]
13.	TiO_2_, ZnO, GO-Fe_3_O_4_	UV light irradiation 18 W, AMX 15 mg/L, pH 5	27.6 48.6 87.1	0.25 0.25 0.25	[29]
14.	CaTiO_3_ (0.5 g) and NaCl (0.06 g) as electrolytes	UV light irradiation, intensity 15 W, photoelectrocatalytic, 200 mL AMX 100 mg/L, pH 3, 45 °C	79	2	[30]

### 2.2. Non-TiO_2_-Based Catalysts

Many researchers have used non-TiO_2_-based catalysts for the photodegradation of AMX. The studies reported in this section are summarized in Table 2.

Gadore et al. synthesized a novel integrated photocatalytic adsorbent by employing tea leaf extract (biochar) as a stabilizing agent and SnS_2_ as a photocatalyst for AMX photodegradation [35]. The overall removal efficiency of AMX was 93.7% achieved within 90 min. Ramos et al. synthesized Fe NPs using pumpkin (Tetsukabuto) peel extract and used them for the photocatalytic degradation of AMX under UV light irradiation [36]. The optimum conditions achieved for the 60% AMX degradation were 2.5 g/L of Fe NPs, initial AMX concentration 10 mg/L, pH 5.6, and 60 min irradiation time. Mmelesi et al. synthesized Zn_x_Co_1−x_Fe_2_O_4_ (x = 0.0 to 0.5) NPs by co-precipitation and calcination [37]. The performance of the prepared NPs was 89% for AMX photodegradation using visible light irradiation. 

**Figure 15:**
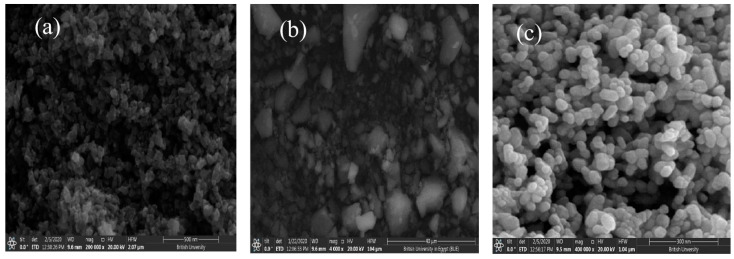
SEM images of photocatalysts (**a**) MIL-53(Al)/ZnO, (**b**) MIL-53Al, and (**c**) ZnO [38].

**Figure 16:**
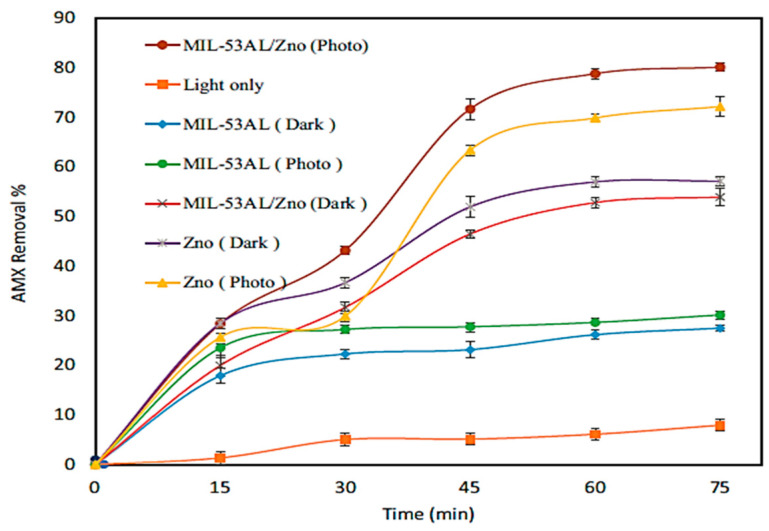
Photodegradation results of AMX using different catalysts, using 50 mg/L of AMX and 0.6 g/L of catalyst at pH 7.0 [38].

**Figure 17:**
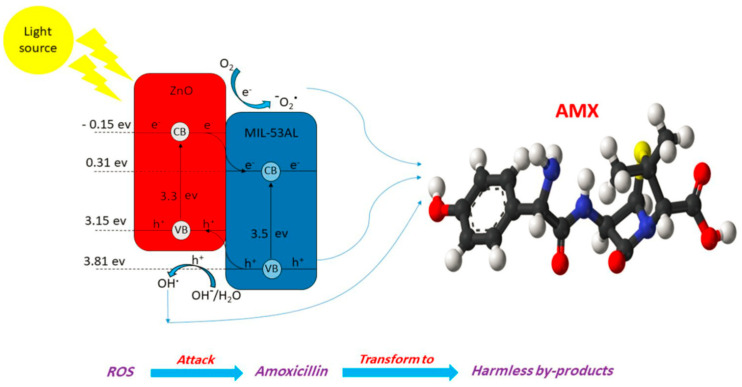
The charge transfer process between ZnO and MIL-53Al and the degradation routes of AMX [38]. AMX molecule has Red: Oxygen, Blue: Nitrogen, Yellow: Sulfur, White: Hydrogen, Black: Carbon.

**Figure 18:**
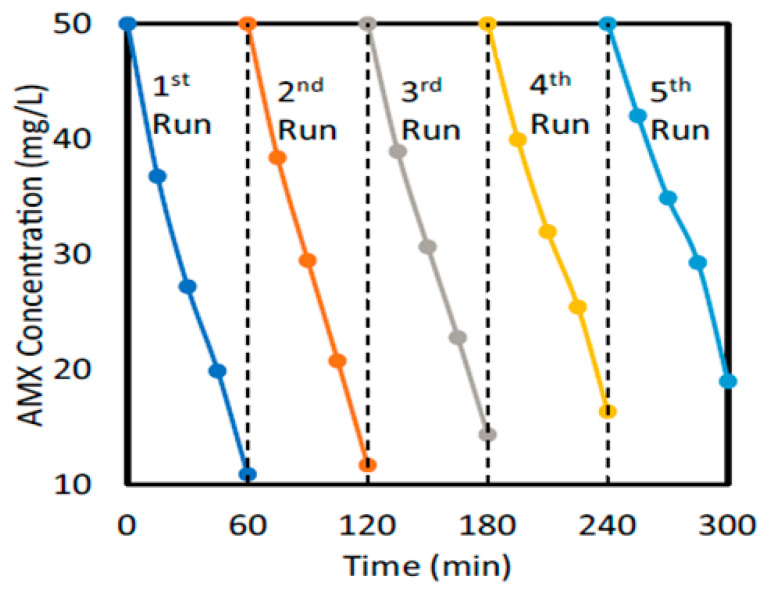
Recyclability results of AMX in five runs using 0.6 g/L of MIL-53(Al)/ZnO catalyst at pH 7.0, 50 mg/L of AMX, and irradiation time of 60 min [38].

Asmaa et al. synthesized MIL-53(Al)/ZnO NPs for the photocatalytic degradation of AMX and utilized them under visible irradiation using a metal halide lamp [38]. Figure 15 shows the SEM photographs to illustrate the morphology of the MIL-53(Al)/ZnO, MIL-53Al, and pure ZnO. Figure 16 shows the degradation of AMX under different catalysts and confirms that the highest degradation was obtained using MIL-53(Al)/ZnO NPs. The removal of AMX was 100% within 60 min of irradiation at pH 4.5 using the catalyst dose of 1.0 g/L and initial AMX concentration of 10 mg/L. The charge transfer process between ZnO and MIL-53Al, and the degradation mechanism of AMX, are shown in Figure 17. The AMX degradation was decreased after five consecutive cycles from 78.2 to 62.1% using MIL-53(Al)/ZnO, as shown in Figure 18. The recyclability tests were conducted using 0.6 g/L of catalyst at pH 7.0, 50 mg/L of AMX, and an irradiation time of 60 min.

**Figure 19:**
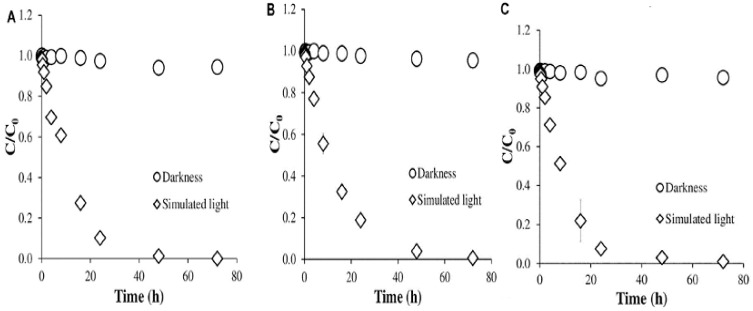
Kinetics plots of AMX degradation at (**A**) pH 4.0, (**B**) pH 5.5, and (**C**) pH 7.0 [39].

**Figure 20:**
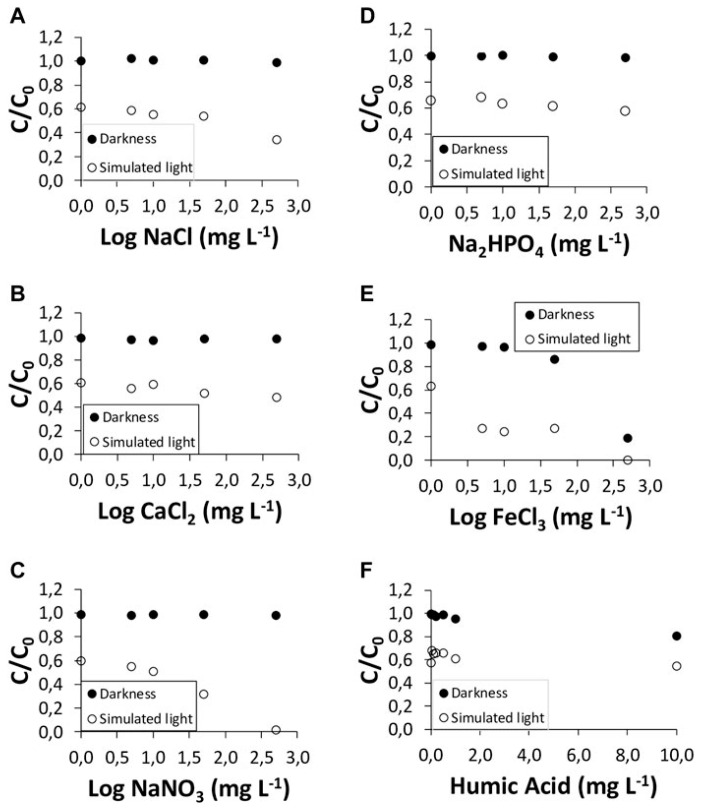
AMX degradation under simulated sunlight and in the dark using inorganic salts: (**A**) NaCl; (**B**) CaCl_2_; (**C**) NaNO_3_; (**D**) Na_2_HPO_4_; (**E**) FeCl_3_; and (**F**) humic acids [39].

Figure 19 shows the degradation kinetics of AMX at pH 4.0, 5.5, and 7.0. There was no degradation of AMX in the dark at all three pH, while almost complete degradation occurred under simulated sunlight, after 40 h of irradiation. Figure 20 shows AMX degradation under simulated sunlight and in the dark in the presence of humic acids and different inorganic salts. In the presence of FeCl_3_ at concentrations of 500 mg/L, AMX showed total degradation, both under simulated sunlight and in the dark [39].

Utami et al. synthesized Bi_2_O_3_/Fe by a precipitation method using microwaves [40]. The best-performing sample was 3% Fe/Bi_2_O_3_, which provided 76.34% degradation efficiency of the AMX.

Le et al. synthesized vanadium pentoxide/graphitic carbon nitride and achieved 91.3% AMX degradation under solar light [41].

Thuan et al. prepared indium vanadate and silver deposited on graphitic carbon nitride (InVO_4_@Ag@g-C_3_N_4_) for photocatalytic degradation of AMX, which exhibited high photocatalytic performance and recyclability for AMX degradation [42].

Palas and Ersöz prepared Ag and Co-doped perylene diimide supramolecular catalysts for the photocatalytic removal of AMX from aqueous solutions [43]. The AMX photodegradation was 51.8% at pH 4.6, 0.52 g/L catalyst loading, and 10.3 mg/L AMX concentration. The AMX degradation fits a second-order kinetic model.

Moradi et al. studied CuO NPs and ZnO nanorods supported on g-C_3_N_4_ nanosheets (CZ@T-GCN) as novel photocatalysts for the degradation of AMX [44]. The results demonstrated enhanced degradation of AMX compared with individual components and showed complete degradation of AMX within 120 min of simulated sunlight irradiation using a catalytic dosage of 0.9 g/L at pH 7.0. 

Shi et al. prepared a composite by loading carbon dots onto rod-like CoFe_2_O_4_ that efficiently degraded AMX by 97.5% within 80 min using photocatalytic activation of peroxymonosulfate under visible light irradiation [45].

Xu et al. reported a 2D/2D Bi_2_WO_6_/Ti_3_C_2_ MXene heterostructure that achieved complete degradation of AMX within 40 min [46] due to the generation of reactive oxygen species, mainly holes (h^+^) and superoxide anion radicals (O_2_^−^).

Le et al. fabricated a CdS/NH_4_V_4_O_10_ composite by loading CdS quantum dots into the ultrathin NH_4_V_4_O_10_ nanobelts [47]. The synthesized composite possesses a large surface area and plentiful active sites for photodegradation. The optimal composite, 6-CdS/NH_4_V_4_O_10_, provided 94.4% degradation performance with 5.0 wt% AMX with high recyclability.

Le et al. synthesized carbon dot-loaded Sn_2_Ta_2_O_7_/SnO_2_ (C-dots/Sn_2_Ta_2_O_7_/SnO_2_) heterostructures that exhibited AMX (initial concentration 20 mg/L) photodegradation efficiency of 88.3% within 120 min under simulated solar light irradiation [48].

Dou et al. synthesized mesoporous carbon nitride and used it for the photodegradation of AMX and cefotaxime [49] and achieved excellent performance under visible light.

Samsudin et al. synthesized a g-C_3_N_4_/BiVO_4_ microflower and evaluated it for the photocatalytic degradation of AMX. It was found that 89.5% of the AMX was successfully degraded within 3 h under visible light irradiation [50]. 

Nguyen et al. reported a 100% photodegradation efficiency of AMX under simulated solar irradiation using WO_3_ at pH 4, a catalyst dosage of 0.104 g/L, and initial AMX concentration of 1.0 μM [51]. The photodegradation of AMX was found to follow the pseudo-first-order kinetics.

Chahkandi et al. synthesized thin BiVO_4_ nanorods and deposited them on a webbed stainless-steel surface [52]. The diameter of the BiVO_4_ nanorods was in the range of 100 to 150 nm, and it exhibited 97.5% AMX photodegradation in 1.5 h under visible light irradiation.

Abazari et al. prepared amine-functionalized Al-based nanocomposites (NH_2_-MOF@Sm_2_O_3_–ZnO) and used them efficiently for the photodegradation of AMX under ultrasound and visible light [53]. These nanocomposites showed stable photodegradation performance even after five recycles.

**Figure 21:**
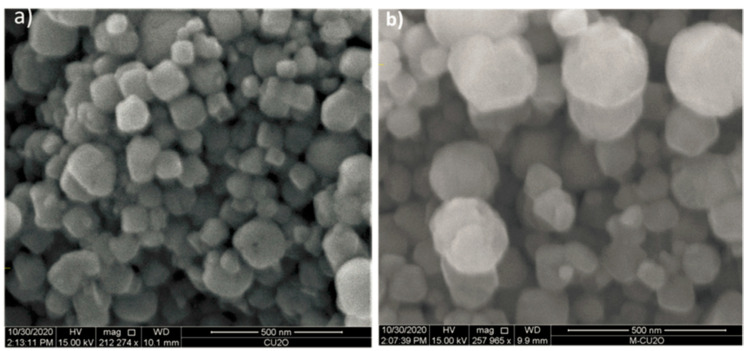
SEM images of (**a**) Cu_2_O and (**b**) Mn-doped Cu_2_O [54].

**Figure 22:**
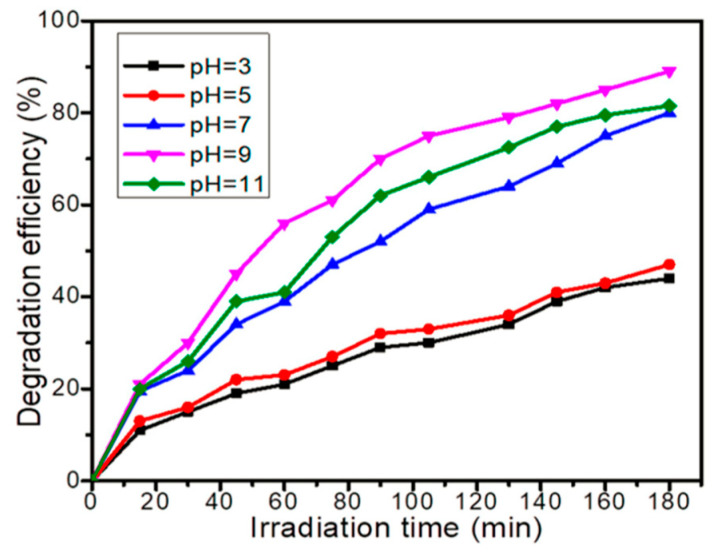
Effect of pH on photocatalytic degradation of 15 mg/L AMX using 1 g/L of Mn-doped Cu_2_O catalyst [54].

**Figure 23:**
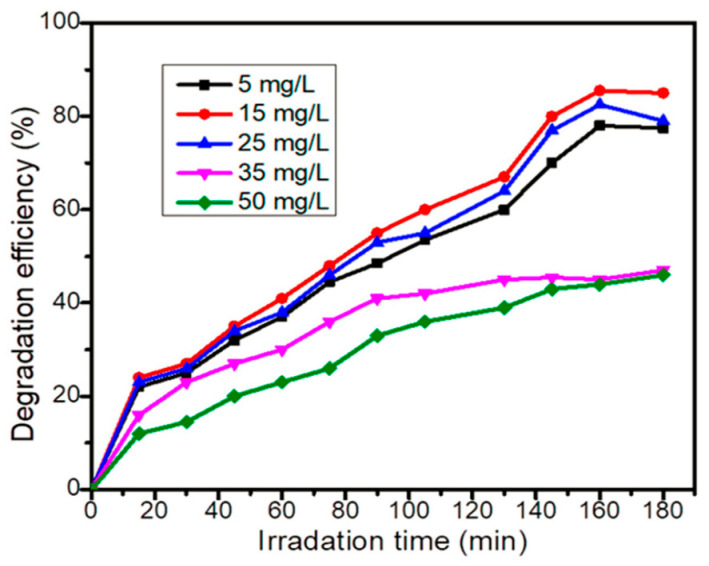
Effect of initial concentration of AMX on photocatalytic degradation using 1 g/L Mn-doped Cu_2_O catalyst at pH 9 [54].

**Figure 24:**
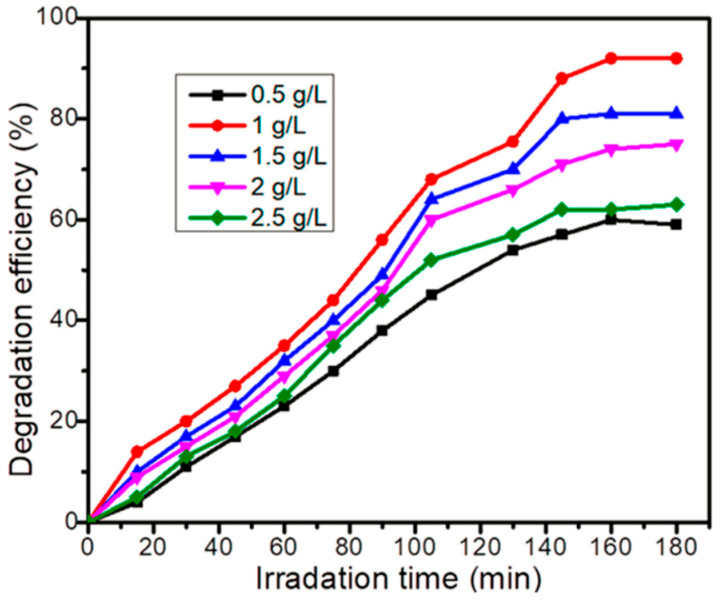
Effect of Mn-doped Cu_2_O catalyst dose on photocatalytic degradation of 15 mg/L AMX at pH 8 [54].

**Figure 25:**
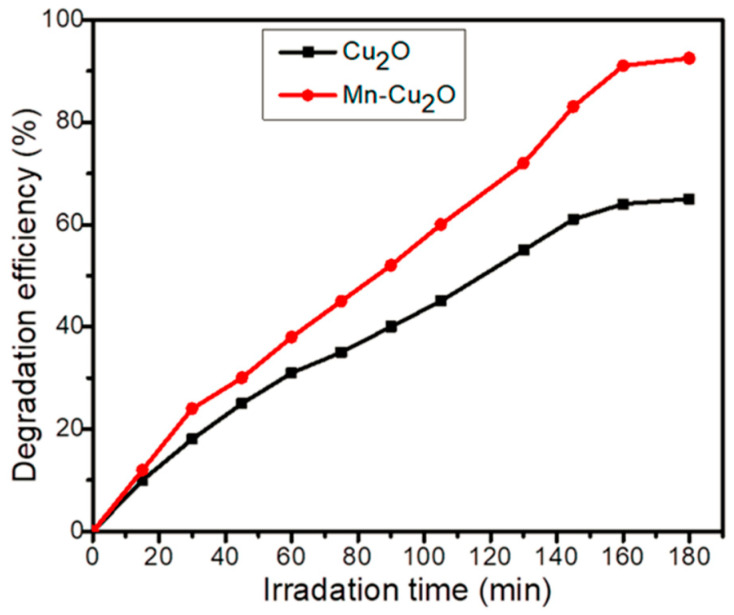
Photodegradation of AMX using Cu_2_O and Mn-doped Cu_2_O under the optimal conditions of 15 mg/L of AMX and 1 g/L of catalyst at pH 9 [54].

Gaim et al. reported the synthesis of Mn-doped Cu_2_O NPs using aloe vera leaf extract and used them for AMX photodegradation [54]. The AMX photodegradation achieved was 92.0% at pH 9, 15 mg/L initial concentration of AMX, and catalyst dosage of 1 g/L under sunlight irradiation. Figure 21 shows the SEM images of pure Cu_2_O and Mn-doped Cu_2_O NP, which reveal that their morphologies were octahedral and truncated octahedral, respectively. Figure 22 shows the effect of pH on the photocatalytic degradation of AMX (initial concentration 15 mg/L) using a catalyst dosage of 1.0 g/L. The degradation of AMX was highest at pH 9 due to the formation of large amounts of OH radicals at higher pH. Figure 23 exhibited the effect of the initial concentration of AMX on the photodegradation of AMX at pH 9 using 1.0 g/L catalyst concentration. The degradation was first increased with increasing AMX concentration up to 15 ppm and then decreased as the AMX concentration increased. Figure 24 shows the effect of catalyst concentration on the photocatalytic degradation of AMX (15 mg/L) at pH 8. Different amounts (0.5 to 2.5 g/L) of the catalyst were added to the AMX solution. Maximum degradation of AMX was observed at 1.0 g/L catalyst dosage. Figure 25 shows the photodegradation of AMX using Cu_2_O and Mn-Cu_2_O conducted at pH 9, using an AMX concentration of 15 mg/L and 1.0 g/L catalyst, which achieved 65.0% and 92.0% degradation, respectively, within 3 h of irradiation. 

The photodegradation of AMX was performed using the photo-Fenton process with an effective irradiation time of 30 min. Nonetheless, AMX photodegradation with photo-Fenton is a costly process [55]. Furthermore, the effect of pH is very substantial in the AMX photodegradation with photo-Fenton [56].

**Figure 26:**
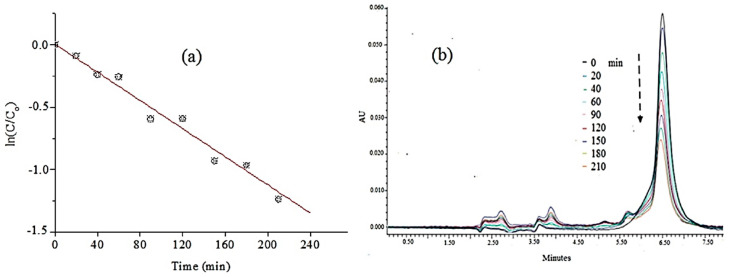
Photodegradation of AMX using (**a**) Ba(Ti_0_._950_Sc_0_._025_Nb_0_._025_)O_3_ and (**b**) the spectra showing degradation with irradiation time [57].

Haddadou et al. synthesized Ba(Ti_0_._950_Sc_0_._025_Nb_0_._025_)O_3_, termed as BTSN, as a tetragonal perovskite structure with a ferroelectric behavior and photoelectrochemical properties having a band gap of 2.60 eV [57]. The BTSN was tested for the photocatalytic oxidation of AMX under solar light irradiation, exhibited 72% conversion, and followed first-order kinetic. Figure 26 shows the photodegradation of AMX and the spectra showing AMX degradation with irradiation time.

Kattel et al. investigated the degradation of AMX by the UVC (280 nm) or UVC/Fe^2+^-activated S_2_O_8_^2−^ processes [58]. The optimal conditions were the concentration of S_2_O_8_^2−^ and Fe^2+^, 15400 μM and 40 μM, respectively, and the reaction followed pseudo-first-order kinetics. AMX degradation achieved was more than 99% in 2 h irradiation time. Both radicals, SO_4_**^•^**^−^ and HO**•**, were found to be present in the reaction system, but SO_4_**^•^**^−^ was in the major amount. 

Beshkar et al. reported employing a CuI/FePO_4_ heterojunction nanocomposite to degrade AMX under simulated solar exposure [59]. The hybrid CuI/FePO_4_ NPs demonstrated an energy gap of 3.03 eV and a photodegradation efficiency of 90%.

Silva et al. synthesized metal-free polymeric carbon nitrides (PCNs) using melamine and thiourea precursors [60] and evaluated them for AMX photodegradation under visible light irradiation (16 mW/cm^2^). The thiourea-based material showed better efficiency of 100% removal within 48 h and excellent recyclability.

Mirzaei et al. investigated a fluorinated graphitic carbon nitride photocatalyst having magnetic properties for the degradation of AMX in water [61]. The photocatalyst exhibited easy separation from the aqueous solution by a magnet and improved photocatalytic degradation of AMX. The study showed that a UV lamp (10 W) was more efficient for AMX degradation than a 500 W visible lamp.

Table 2 lists the reaction conditions and performance of non-TiO_2_-based nanomaterials and nanocomposites employed for AMX photodegradation. These catalysts comprise metal nanoparticles and single and multiple metal and metal oxides derived from molybdenum, zinc, silver, manganese, copper, cerium, bismuth, vanadium, and tungsten. The doping and mixing of the metals were shown to be very effective in the photodegradation of AMX and to have enhanced degradation efficiency.

**Table 2:** Performance of non-TiO_2_-based nanomaterials (NMs) and nanocomposites used for photodegradation of AMX.

No	Catalyst Type	Process Conditions	Degradation Efficiency, %	Degradation Time, h	Ref.
1.	MIL-53(Al)/ZnO nanocomposite, 1.0 g/L	Visible light irradiation, AMX 10 mg/L, pH 4.5	100	1	[39]
2.	Iron NPs (Fe-NPs), 2.5 g/L	UV light irradiation, AMX 10 mg/L, pH 5.6	60	1	[37]
3.	C-dots/Sn_2_Ta_2_O_7_/SnO_2_ nanocomposite	Simulated solar light irradiation, AMX 20 mg/L	88.3%	2	[49]
4.	g-C_3_N_4_/BiVO_4_ microflower	Visible light irradiation	89.5	3	[50]
5.	WO_3_ NPs, 0.104 g/L	Sunlight irradiation, AMX 1.0 μM, 50 mL solution, pH 4	100	3	[51]
6.	WO_3_ NPs, 0.104 g/L	Simulated solar irradiation (300 W xenon lamp), AMX 1.0 μM, pH 4, follows pseudo-first-order kinetics, temp. 25 °C	100	3	[52]
7.	BiVO_4_ thin film	Visible light irradiation	97.5	1.5	[53]
8.	Mn-doped Cu_2_O NPs, 1.0 g/L	Sunlight irradiation, pH 9, AMX 15 mg/L	92	3	[55]
9.	Cu_2_O NPs, 1.0 g/L	Sunlight irradiation, pH 9, AMX 15 mg/L	65	3	[55]
10.	gamma-Fe_2_O_3_@SiO_2_@ZIF8-Ag nanocomposite, catalyst 0.4 g/L	Visible light radiation, pH 5, AMX 10 mg/L	100	1	[62]
11.	1% Na-doped hydroxyapatite, 0.1 g	AMX 20 mg/L, 50 mL solution, pH 7	60	2	[63]

### 2.3. Photolysis Studies

Vettorello et al. [64] investigated the degradation of AMX by photolysis and assessed the formation of breakdown organic structures and their metabolite toxicity. Solutions of 100 µg/L of AMX were subjected to different conditions of UV irradiation with different powers in a quartz batch reactor at pH 4, 7, and 9. The HPLC-MS analysis determined the degradation of AMX and the reaction products generated. The best 90% AMX degradation was achieved using 95 W lamp irradiation located 5–10 cm from the AMX solution within 10 min at pH 9. Two products originating under different pH conditions were identified, namely, AMX penicilloic acid and AMX 4-hydroxyphenilglyl. The resulting solution containing these products was found to have no toxicity. 

Zhang et al. [65] studied the reaction kinetics, degradation routes, and antibacterial activity of AMX in UV/H_2_O_2_ and UV/persulfate (S_2_O_8_^2−^, PS) systems. It was found that UV irradiation alone did not affect AMX degradation. However, adding H_2_O_2_ or PS considerably improved degradation efficiency by producing HO. and SO_4_^−^ radicals. At neutral pH, UV, HO., and SO_4_^−^ contributed 7.3%, 22.8%, and 69.9% to AMX degradation, respectively. The AMX degradation pathways using UV/H_2_O_2_ and UV/PS systems included processes such as hydroxylation, hydrolysis, and decarboxylation. UV/H_2_O_2_ photolysis proved to be more cost-effective than UV/PS for AMX degradation. The antibacterial activity of the AMX solution dropped significantly after UV/H_2_O_2_ and UV/PS treatment, indicating that the AMX breakdown products were not significantly hazardous.

Arsand et al. [66] performed photolysis of AMX and ampicillin surface water samples and analyzed the degradation and the breakdown products by HPLC-MS. The results displayed that the degradation of AMX and ampicillin was almost complete in 48 h using river water, and more than 65 transformation products of amoxicillin and ampicillin were detected and identified. The photolysis kinetics of AMX and ampicillin in river water followed pseudo-first-order kinetics.

Timm et al. [67] focused their study on the photolysis of the four β-lactam antibiotics including AMX, ampicillin, penicillin V, and piperacillin under simulated environmental conditions. It was observed that all the investigated β-lactam antibiotics were degradable by simulated sunlight (1 kW/m^2^). Structure interpretation of the transformation products achieved using HPLC-MS showed that the hydrolysis of the β-lactam ring was the primary transformation reaction, followed by the elimination of carboxylic and dimethyl thiazolidine carboxylic acid. The loss of bactericide activity of the irradiated solutions of amoxicillin, ampicillin, and piperacillin suggested that the transformation of the β-lactam ring is responsible for the antibiotic effect of these antibiotics.

## 3. Perspective from Reviews Published Earlier in This Area

Pirsaheb et al. developed a systematic review of the photo-Fenton process for aqueous AMX degradation, revealing that its efficiency depends on factors like pH, light source characteristics, and AMX concentration [68]. Acidic pH values show the highest efficiency. Energy-efficient light sources reduce catalyst and oxidizer use. Combining AOPs like photo-Fenton, electro-Fenton, and photo-electro-Fenton increases mineralization efficiency. Synthetic wastewater shows higher efficiency.

Qutob et al. published a review of radical and non-radical degradation of AMX using different oxidation process systems [69]. The review explores degradation mechanisms, efficiency, catalyst stability, AMX byproduct formation, and toxicity. Pharmaceutical compounds like AMX are increasingly being studied for their potential environmental leakage. Due to its low metabolic rate, 80–90% of AMX remains unmetabolized. Advanced oxidation processes, including photocatalytic, ultrasonic, electro-oxidation, and partials, are effective in degrading AMX. High pH, temperature, concentration, oxidants, catalysts, and doping ratios can inhibit degradation. Hybrid systems like photo-electro, photo-Fenton, and electro-Fenton are recommended for successful oxidation. This review could provide valuable insights for future researchers.

Aryee et al. reviewed the detection and removal of AMX in wastewater, discussing its entry into the environment and potential effects [70]. They discussed detection methods and remediation techniques, with advanced oxidation processes and constructed wetlands being the most and least applied methods. The review also highlighted research gaps and recommendations for further studies on AMX removal, aiming to inspire further research.

Manikanika et al. reviewed the activity of ZnO NPs for dyes and drug photodegradation [71]. Several metal oxides performed as photocatalysts, but ZnO was found to achieve total degradation and mineralization. ZnO NPs have a large band gap that is 3.37 eV and 60 meV excitation binding energy and have performed very well in photocatalysis. The reported maximum degradation efficiency using ZnO NPs has been reported at 100% and 98% for dyes and drugs, respectively. 

Chen et al. reported that antibiotics have been extensively detected in the aquatic environment as one of the major pollutants [72]. For the elimination of antibiotics, photocatalysis using sunlight is considered a promising means because it is environmentally friendly and cost-effective. This review presented a summary of the recent progress in the removal of antibiotic pollutants using the photocatalytic oxidation process within the last five years. The review introduced the general characteristics and environmental dangers of common antibiotics, the basic mechanism of photocatalytic degradation, and the degradation of antibiotics by photocatalysts. Finally, opportunities and challenges in the photocatalytic degradation of antibiotics were discussed. 

Asih et al. presented a review on the use of TiO_2_ photocatalysts for the degradation of dyes and drugs as organic pollutants in aqueous solutions [73]. It was reported that increasing the concentration of the TiO_2_ catalyst and the irradiation time increases the photodegradation of antibiotics. 

Sodhi et al. published a review on the resistance, ecotoxicity, and remediation strategies used for AMX [74]. The increasing use of antibiotics, particularly AMX, has led to water contamination and a global medical crisis. AMX, a common antibiotic used in human and veterinary medicine, is refractory to degradation and cannot be completely removed from the environment. Wastewater treatment plants are struggling to efficiently remove AMX, which is present in drinking water and water bodies. Various methods, including physicochemical parameters, nanoparticles, phytoremediation, and the use of bacteria and algae, are being explored for better and sustainable technology.

## 4. Conclusions

This study presented a review of the published research for the elimination of antibiotic AMX by photodegradation using UV, visible, and solar irradiation in the presence of a variety of NMs. Most of the researchers have used TiO_2_ as the base material, doped and combined with other metals and metal oxides to achieve degradation using visible light. This review was made using research published during the last five years, from 2018–2024. Researchers have used a variety of reaction conditions that include radiation types (UV, solar, and visible), pH of the solution, the concentration of AMX and NMs, presence of other additives such as H_2_O_2_ as oxidants, and different salts like NaCl and CaCl_2_ to achieve high photodegradation efficiency. TiO_2_ was the best nanomaterial found that achieved the highest degradation of AMX in ultraviolet irradiation. TiO_2_ doped with other nanomaterials showed very good performance under visible light. WO_3_ was also used by several investigators and found quite effective for AMX degradation. Other metal oxides used for AMX elimination contain molybdenum, zinc, manganese, copper, cerium, and silver or a combination of these metals. Some researchers have used photolysis of AMX with UV, solar, or visible irradiation in the absence of solid catalysts with or without adding additives and oxidants such as H_2_O_2_.

## Data Availability

The data presented in this study are available from the corresponding author upon reasonable request.
